# Dropsonde observations during the Aerosol Cloud meTeorology Interactions oVer the western ATlantic Experiment

**DOI:** 10.1038/s41597-023-02647-5

**Published:** 2023-11-01

**Authors:** Holger Vömel, Armin Sorooshian, Claire Robinson, Taylor J. Shingler, Kenneth Lee Thornhill, Luke D. Ziemba

**Affiliations:** 1 National Center for Atmospheric Research, Boulder, CO 30301 USA; 2 Department of Chemical and Environmental Engineering, University of Arizona, Tucson, AZ 85721 USA; 3 NASA Langley Research Center, Hampton, VA 23681 USA

**Keywords:** Atmospheric chemistry, Atmospheric dynamics

## Abstract

The Aerosol Cloud meTeorology Interactions oVer the western ATlantic Experiment (ACTIVATE) field campaign provides accurate data for aerosol characterization and trace gas profiles, and establishes knowledge of the relationships between aerosols and water. The dropsonde dataset provides an *in situ* characterization of the vertical thermodynamic structure of the atmosphere during 165 research flights by NASA Langley’s King Air research aircraft between February 2020 and June 2022 and four test flights between December 2019 and November 2021. The research flights covered the western North Atlantic region, off the coast of the Eastern United States and around Bermuda and covered all seasons. The dropsonde profiles provide observations of temperature, pressure, relative humidity, and horizontal and vertical winds between the surface and about 9 km. 801 dropsondes were released, of which 796 were processed and 788 provide complete profiles of all parameters between the flight level and the surface with normal parachute performance. Here, we describe the dataset, the processing of the measurements, general statistics, and applications of this rich dataset.

## Background & Summary

The Aerosol Cloud meTeorology Interactions oVer the western ATlantic Experiment (ACTIVATE) responds to a critical need to improve understanding of aerosol-cloud interactions, which account for the largest uncertainty in total anthropogenic radiative forcing estimates^1,2^. Past field campaigns have been limited in data collection statistics, sampling a narrow range of aerosol and meteorological conditions, and have had insufficient resources to characterize properties in different regions of a vertical column simultaneously. ACTIVATE addresses these issues by acquiring for the first time detailed, simultaneous, and systematic measurements of aerosols and clouds from *in situ* and remote sensing instruments deployed on two coordinated aircraft over multiple years and seasons^3^. The region of study is the western North Atlantic Ocean, which affords a wide range of aerosol^4^ and meteorological/cloud conditions^5^ that are needed to constrain the magnitude of how aerosols affect clouds and, vice versa, how clouds affect aerosols across a wide range of cloud types and meteorological regimes. The three main ACTIVATE objectives are as follows^3^: (i) quantify relationships between aerosol number concentration, cloud condensation nuclei (CCN) concentration, and cloud drop number concentration, and reduce uncertainty in model parameterizations of cloud droplet activation; (ii) improve process-level understanding and model representation of factors that govern cloud micro/macro-physical properties and how they couple with cloud effects on aerosol; and (iii) assess advanced remote sensing capabilities for retrieving aerosol and cloud properties related to aerosol-cloud interactions.

The flight strategy consists of two NASA Langley Research Center aircraft flying in coordination. More specifically, a HU-25 Falcon conducted measurements from near the ocean surface (i.e., approximately 150 m) up to an altitude of 3 km to characterize trace gas, aerosol, cloud, and meteorological state variables below, in, and above boundary layer clouds. A King Air research aircraft simultaneously flew overhead at around 9 km in close columnar proximity to the Falcon. The King Air carried two remote sensors, the Research Scanning Polarimeter (RSP) and the High Spectral Resolution Lidar (HSRL-2), both looking down and characterizing aerosol and cloud parameters. Furthermore, the King Air research aircraft launched National Center for Atmospheric Research (NCAR) NRD41 dropsondes to obtain a detailed vertical distribution of pressure, winds, temperature, and relative humidity (RH).

This data paper describes the observations, which were taken during all observation periods and test flights between December 2019 and June 2022 using the NCAR NRD41 dropsondes.

The dropsonde dataset described here has already been used in recent ACTIVATE publications^6,7^. Dropsonde data help characterize meteorological conditions and derive large-scale divergence, vertical velocities, and surface heat fluxes useful for intercomparison and validation when compared to common reanalysis data sources, e.g., the fifth-generation European Centre for Medium-Range Weather Forecasts Re-Analysis (ERA5), the Modern-Era Retrospective Analysis for Research and Applications, version 2 (MERRA-2), and models using large eddy simulation^7–9^. A subset of ACTIVATE studies have classified wintertime flights into whether they qualify as a “cold air outbreak”^6,10,11^, for which dropsonde data are used along with published criteria requiring knowledge of the vertical thermodynamic structure of the atmosphere^12^. As demonstrated by another recent campaign investigating aerosol-cloud interactions (CAMP^2^Ex), dropsonde data such as vertically-resolved relative humidity can be used for model simulations of aerosol extinction and aerosol optical thickness^13^ and for assistance in interpreting remote sensing data from HSRL-2 and RSP deployed on the King Air. Investigations and analyses of ACTIVATE observations will show how dropsonde data are useful for assessments and improvements in remote sensing algorithms for geophysical variables such as wind speed, mixed layer height, and aerosol-related parameters.

## Methods

### ACTIVATE details

**Fig. 1:**
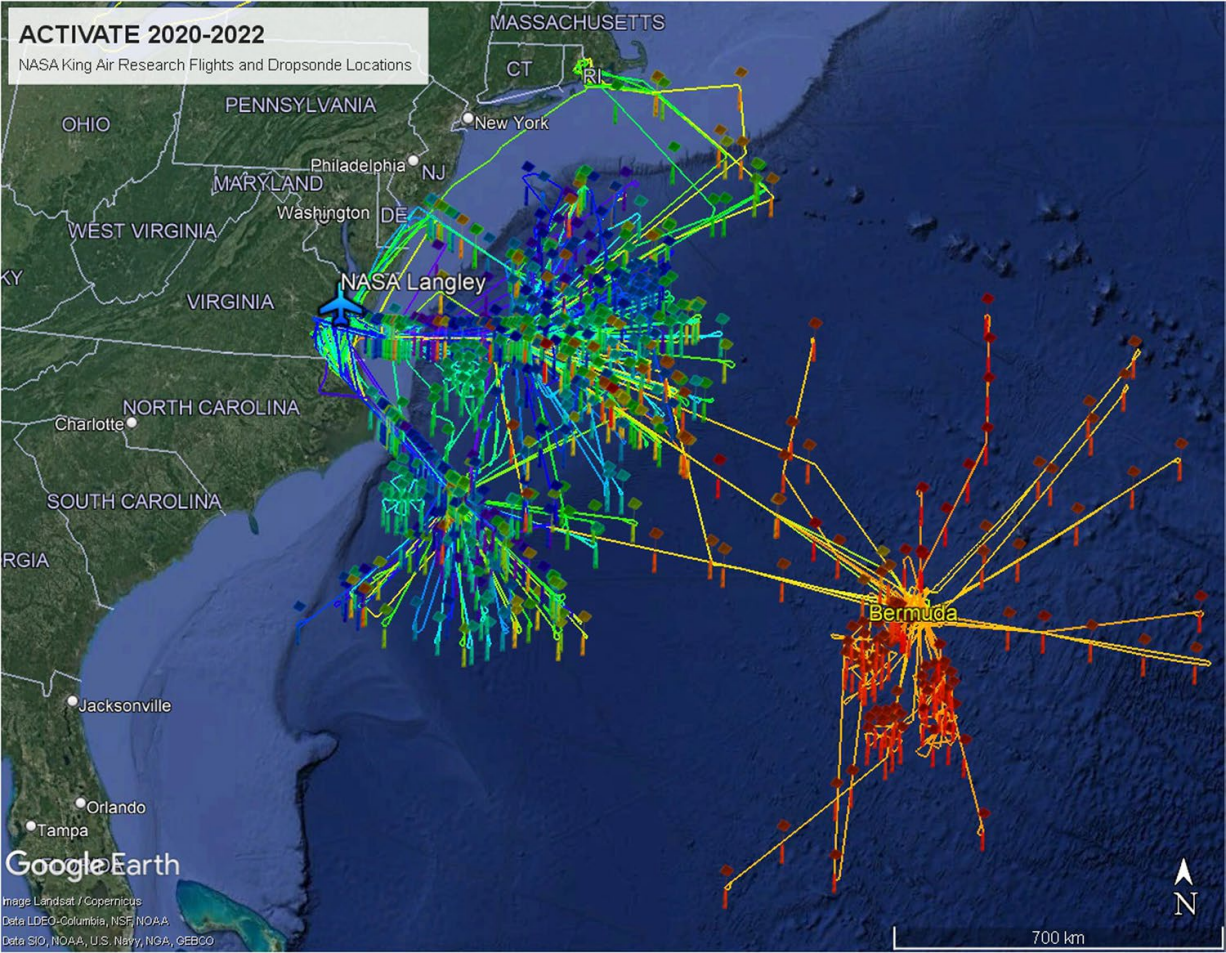
All NASA King Air flight tracks and dropsonde locations during ACTIVATE from February 2020 through June 2022. The flights originating from Bermuda took place in June 2022.

Across the six ACTIVATE deployments, the King Air flew 165 research flights between 14 February 2020 and 18 June 2022, during which 785 dropsondes were successfully released. In addition, the dataset contains the data from the test flights on 16 December 2019, 11 August 2020, 20 January 2021, and 19 November 2021, during which 11 dropsondes were released. Research flights took-off and landed at the NASA Langley Air Force Base, except for the flights in June 2022, which originated at Bermuda (L.F. Wade International Airport). Four flights (9 December 2021, 27 January, 26 February, and 5 May 2022) stopped for refueling at Rhode Island airports before returning to Langley. All flight tracks of the King Air research aircraft are shown in Fig. 1. These flights were typically coordinated with lower altitude flights by the HU-25 Falcon, which are not shown.

Research flights typically lasted between three and four hours and were conducted at different times of the year to represent different pollution, weather, and cloud characteristics. Take off times were typically in the local early and late afternoon with one or two flights per campaign day.

Table 1 provides an overview of all research flights during which dropsondes were released. Table 2 provides an overview of the success statistics for all soundings launched during the campaign, including all test sondes. Out of 801 sondes, four (0.50%) failed to detect launch and the sensor module failed prior to launch in one sonde. These soundings did not provide useful data and were ignored. Out of the remaining 796 soundings, three soundings (0.38%) did not provide data down to the surface. In four sondes, the GPS module failed after launch and in one sounding the parachute failed after it was successfully released. 788 soundings provided complete profiles of all parameters under normal parachute performance, i.e., the system performed with a 98.4% success rate. Some minor issues were encountered, which were rectified in post processing. The quality control procedures are described in detail in the following sections.

**Table 1:** Overview of all successful sonde releases during ACTIVATE. Note that the flight numbering for the King Air aircraft is not contiguous and ignores flights by the HU-25 Falcon, which did not release dropsondes. A * following the number of dropsonde launches indicates research flights containing soundings with at least one issue discussed in the following paragraphs.

**Table 2:** Overview of the dropsonde system performance.

	# of Sondes	Percent
Total number of sondes released	801	100
Sondes processed	796	99.4
Complete wind and thermodynamic profiles to the surface with normal parachute performance	788	98.4

### AVAPS dropsonde sounding system

The NCAR AVAPS dropsonde system deployed in ACTIVATE used the manual dropsonde launch tube onboard the King Air research aircraft and the NCAR Research Dropsonde model NRD41. This dropsonde uses the pressure, temperature, and humidity sensor of the Vaisala RS41 radiosonde and employs an improved version of the GPS, telemetry, and parachute release system of the previous NRD94 dropsonde^14^, which was in use between 2011 and 2018. The NRD41 dropsondes have been successfully deployed during the Organization of Tropical East Pacific Convection (OTREC) in August and September 2019^15^.

NCAR developed the smaller NRD41 dropsonde in parallel with its larger version, the RD41 dropsonde. This larger version has been introduced into operational service by NOAA and the Air Force in 2018 and is commercially produced and marketed by Vaisala. The reliability of the sensor measurements can be considered equivalent between both types. The largest functional difference is the launch procedure and parachute release. While the larger RD41 is used exclusively in manual dropsonde launchers, the smaller NRD41 can be used in manual and automated dropsonde launchers. In addition, the NRD41 uses a parachute release mechanism, which is electronically controlled and triggered after launch of the sonde. This method is more reliable than the mechanical delay ribbon used on the larger RD41, leading to far fewer launch-detect and fast-fall problems.

The NRD41 and RD41 (in short xRD41) dropsondes make use of the heated humidity sensor of the Vaisala sensor unit, which eliminates common bias and icing problems in humidity measurements. Temperature is measured by a platinum-wire sensor, pressure is measured by a solid-state pressure transducer, and position and velocity are measured by Global Navigational Satellite System (GNSS) positioning.

Two out of eight drops during the initial test flight on 16 December 2019 used the older NRD94 dropsonde model. The temperature and humidity measurements are of slightly lesser quality than those of the NRD41 sondes. The NRD94 soundings are not discussed further.

The AVAPS LabVIEW based data acquisition software (version 4.1.2) received and stored data from the dropsondes, the aircraft data system, and the local AVAPS GPS unit.

The manual dropsonde launch tube was installed towards the back of the NASA King Air and was operated by a flight scientist to launch the dropsondes.

### Standard quality control

Standard quality control (QC) in near real time and as part of the final data QC is based on the algorithms implemented in the ASPEN software (https://www.eol.ucar.edu/content/aspen). The following quality checks, corrections, and calculations are performed by ASPEN (version 3.4.7):

- Removal of outliers and suspect data points in pressure, temperature, humidity, zonal and meridional wind, latitude, and longitude
- Removal of data between release from the aircraft and equilibration with atmospheric conditions
- Dynamic correction to account for the lag of the NRD41 temperature sensor using the appropriate coefficients for the NRD41 dropsondes
- Dynamic correction to account for the sonde inertia^16^ in the determination of the wind profile using the appropriate parameters for the NRD41 dropsondes
- Smoothing of pressure, temperature, humidity, zonal and meridional wind using a B-spline algorithm
- Recomputing of wind speed and wind direction after smoothing of the wind components
- Extrapolation of the last reported pressure reading to a surface pressure value (where possible), based on the fall rate of the sonde
- Recalculation of the geopotential height from the surface to the top of the profile
- Computing a vertical wind velocity component

The equilibration time for the temperature and RH sensor was adjusted to 10 s to remove data influenced by the release from the aircraft. The smoothing time for all parameters was adjusted to 5 s.

### Additional quality control

All soundings were carefully investigated for any minor issue, which could not be handled by the standard QC using ASPEN. The following sections describe the performance of the mechanical and measurement system components, and the relevant corrections applied that were not captured by ASPEN. We also describe all occasions where not all components of the instrument worked as intended. Data were set to missing if they showed obvious inconsistencies and were otherwise left in place.

### Missing real-time data

A synchronization error between the onboard computer and the GPS time led to loss in real-time processed data during 18 soundings. This problem occurred only in flights where the clock of the receiving computer had inadvertently not been set properly. Depending on the details of the synchronization, the real time display of data may have indicated a complete loss of telemetry or in some cases a loss of only a small number of data lines. Storage of raw data was not affected by this error and all raw data received were stored independent of the accuracy of the computer clock as long as the operator kept the receiving channel open.

The soundings affected by loss of real time data, which were recreated in post processing, are listed in Table 3.

**Table 3:** Soundings during which real time data were not available.

#	Research Flight	Sounding	Missing data lines
1	RF01	20200214_224743	477
2	RF01	20200214_232630	689
3	RF01	20200215_002529	281
4	RF09	20200227_183431	1125
5	RF09	20200227_195629	1109
6	RF32	20200910_174604	1079
7	RF32	20200910_182712	1125
8	RF37	20200922_181332	29
9	RF39	20200929_154230	24
10	RF39	20200929_160608	30
11	RF67	20210519_140632	138
12	RF84	20210616_141625	94
13	RF84	20210616_150157	1180
14	RF84	20210616_152603	937
15	RF130	20220302_202730	1201
16	RF130	20220302_203314	854
17	RF130	20220302_210901	1279
18	RF130	20220302_222514	1296

Missing data lines were recovered from raw data in post-processing.

### Incorrect launch time

Related to the synchronization error, 17 data files were stored with an incorrect launch time, which may have been reported off by as much as five hours. In these files, the launch date and time were corrected using the correct time contained in the raw binary data. The files for which the launch time was changed are listed in Table 4. Note that for tracking processed and raw data, the file names were not changed. The correct launch date and time are coded in the metadata contained in each file and will be in conflict with the file names. The date and time stamps contained in the file names should not be decoded to identify the correct launch date and time.

**Table 4:** Soundings with an incorrect time stamp.

#	Research Flight	Sounding	Correct launch time	Time correction	Notes
1	RF01	20200214_222931	20200214_172927	−5 hrs	
2	RF01	20200214_224743	20200214_174738	−5 hrs	Data in the top 200 m are missing
3	RF01	20200214_232630	20200214_182627	−5 hrs	
4	RF01	20200215_002529	20200214_192525	−5 hrs	
5	RF09	20200227_183431	20200227_183519	48 sec	
6	RF09	20200227_195629	20200227_195739	70 sec	
7	RF32	20200910_174604	20200910_174711	67 sec	
8	RF32	20200910_182712	20200910_182907	115 sec	
9	RF67	20210519_140632	20210519_140730	58 sec	fast fall with missing launch detect
10	RF84	20210616_141625	20210616_151625	1 hrs	
11	RF84	20210616_150157	20210616_160337	1 hrs	
12	RF84	20210616_150541	20210616_160543	1 hrs	
13	RF84	20210616_152603	20210616_162603	1 hrs	
14	RF84	20210616_163148	20210616_173148	1 hrs	
15	RF130	20220302_202730	20220302_202858	88 sec	
16	RF130	20220302_210901	20220302_210959	58 sec	
17	RF130	20220302_222514	20220302_222601	47 sec	

Related to the incorrect launch times, the metadata of the original files may have reported an incorrect drop location or no location at all. Along with correcting the launch time, we updated the metadata for the location of the drop using the other available aircraft data streams. All data in the archive report the correct date, time, and location.

### Pressure

The pressure sensors used in the NRD41 dropsondes are identical to those used in the Vaisala RS41 radiosondes. Unlike the radiosondes, where a one-point recalibration of the sensor is done prior to launch, here, the pressure sensor is recalibrated during the production of the dropsondes, leading to very low biases.

The mean bias correction applied during production for all ACTIVATE sondes was −0.99 ± 0.34 hPa and only slightly smaller than the calibration bias correction during OTREC^15^. In four sondes (Table 5), the bias correction had not been properly stored, and these sondes were launched without this correction. It was applied in post-processing using the information generated during production.

**Table 5:** Soundings for which the missing pressure offset correction was applied in post-processing.

#	RF	Sounding	Pressure Offset [hPa]
1	RF28	20200826_160958	0.99
2	RF40	20200930_163542	1.14
3	RF45	20210210_174535	1.21
4	RF161	20220531_133514	0.88

During ACTIVATE, 741 sondes occasionally duplicated a reported pressure measurement. This happened up to 27 times per sounding with a median of eight repetitions. While this is barely noticeable in any vertical profile, it did cause additional noise in the calculated vertical fall rate and derived vertical winds. These duplicated pressure readings were interpolated, and the fall rates recalculated in post processing. Only pressure readings had to be corrected. Temperature and relative humidity readings did not show any artificial duplication of measurements. The source of the pressure repetition has meanwhile been attributed to a firmware bug inside the NRD41 and RD41 dropsondes and a fix for this issue is currently under validation. A correction for this pressure duplication is scheduled to be implemented in ASPEN.

### Temperature

The calibration of the temperature sensors was validated during production of the dropsondes and agreed to within 0.2 K with a reference sensor with a two-sigma confidence level (k = 2). With response times much less than 1 s, these profiles allow the highest vertical resolution of temperature measured by dropsondes. During the campaign, all soundings but two showed consistent temperature observations within expected limits.

Sounding 20200922_181332 (RF37) showed an unusual equilibration of the temperature sensor after launch. In this sounding, temperature data were set to missing during the first minute of the drop, leading to a loss of data in the top 800 m of the profile between 7.9 and 8.7 km. Sounding 20210526_143838 (RF73) showed unexplained temperature cold spikes in the top 2 km of the profile. Temperature data above 6.8 km altitude was set to missing. The temperature profile below that appeared normal.

### Relative humidity

The calibration of the humidity sensors was validated during production at 71% RH. The sensors agreed to within 1.7% RH with their reference (k = 2). To achieve this level of confidence in atmospheric observations, the RH sensor on the xRD41 dropsondes need to be reconditioned prior to launch. This reduces the potential of sensor contamination to a minimum and assures the best measurement performance throughout the entire altitude and temperature range of the profiles.

The dedicated heating cycle of the sensor prior to launch reconditions the sensor material and restores the original calibration. After successful reconditioning, the humidity sensors are expected to perform with negligible calibration drift. The sondes store the information whether the reconditioning was successful and allow verification of proper reconditioning prior to take off.

In 20 soundings (Table 6), the relative humidity sensor was not successfully reconditioned prior to take off. Absorption of contaminants into the sensor material due to outgassing by packaging materials and other unidentified sources are likely to degrade the calibration of the sensor during this time. The accuracy of humidity measurements of these sondes is degraded and these soundings are likely to exhibit a small dry bias.

**Table 6:** Soundings in which the humidity sensor was not reconditioned before flight.

#	RF	Sounding
1	Test	20191216_184052
2	Test	20191216_191320
3	Test	20191216_192040
4	Test	20191216_192316
5	RF02	20200215_172514
6	RF02	20200215_185506
7	RF20	20200311_134234
8	RF64	20210514_210237
9	RF77	20210602_184503
10	RF77	20210602_185004
11	RF80	20210607_184023
12	RF80	20210607_193522
13	RF90	20210628_143502
14	RF94	20211130_171557
15	RF94	20211130_174337
16	RF96	20211207_185240
17	RF161	20220531_133514
18	RF171	20220610_180657
19	RF173	20220611_181926
20	RF173	20220611_193332

### GPS performance

The dropsonde GPS receivers operated in all but four soundings. The average speed uncertainty reported by the GPS was around 0.2 m/s. This speed uncertainty reflects the confidence of the horizontal speed determined by the algorithm of the GPS unit. Fifteen soundings (Table 7) had a slightly degraded performance with a speed uncertainty of 1.0 m/s. ASPEN had been configured to remove the wind measurements under these conditions and real time processed data missed wind information in these soundings. In post-processing, the thresholds were increased for the affected soundings, recovering the wind measurements that had been rejected in real time. We estimate that this increases the uncertainty in wind measurements by possibly up to 0.8 m/s, which is still acceptable for all scientific studies.

**Table 7:** Soundings in which the GPS accuracy was slightly degraded.

#	RF	Sounding
1	RF60	20210402_144456
2	RF85	20210617_154436
3	RF97	20211209_135651
4	RF98	20211209_174235
5	RF104	20220115_151644
6	RF106	20220118_194932
7	RF115	20220201_160932
8	RF118	20220203_194904
9	RF126	20220222_162620
10	RF131	20220303_145934
11	RF135	20220307_151005
12	RF149	20220503_151501
13	RF170	20220610_125436
14	RF170	20220610_145109
15	RF171	20220610_195639

In four soundings (Table 8), the GPS module failed completely, and no wind measurements were reported in the archive for these profiles.

**Table 8:** Soundings where the GPS module failed.

#	RF	Sounding
1	RF97	20211209_154136
2	RF103	20220112_193337
3	RF104	20220115_151358
4	RF105	20220118_142138

### Parachute performance

**Fig. 2:**
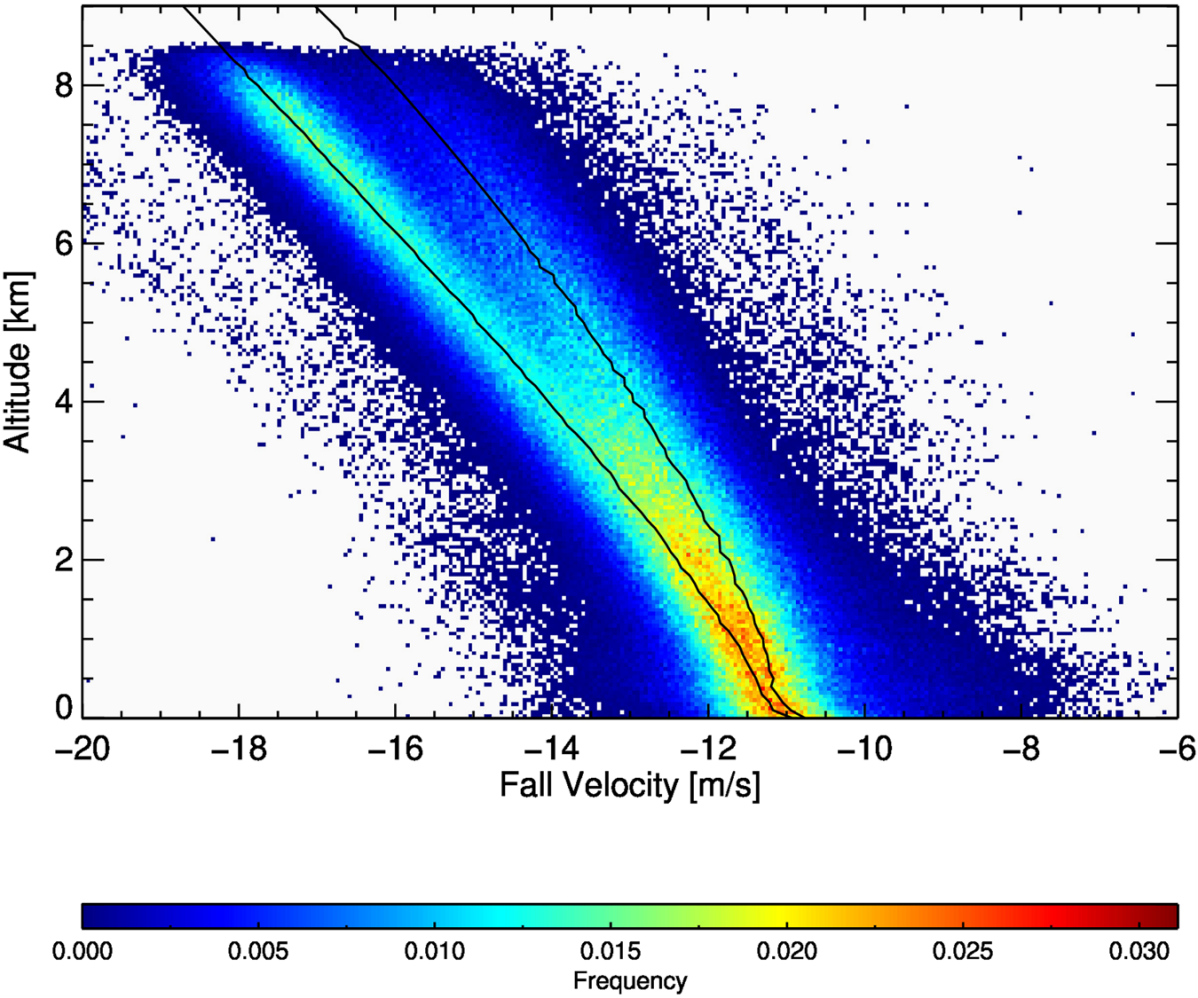
Distribution of fall velocities during ACTIVATE. A fit for the two different parachute models is indicated by the black lines.

The parachutes performed within expected limits in 794 soundings and failed in six soundings. The parachutes in dropsondes used during ACTIVATE were produced by two different manufacturers using the same specifications. Despite being built following identical specifications, the average fall rates for parachutes from each manufacturer show small but systematic differences. The distribution of fall rates (Fig. 2) shows a bifurcation in the middle troposphere. The variability in this distribution is due to the inherent noise of determining the fall rate from measurements of pressure, temperature, and humidity, and due to the variability of the parachute production and atmospheric vertical motion.

Figure 2 includes a fit to the mean fall rate for each of the two different parachute models. In the middle troposphere the fall rate difference is about 1.5 m/s at 6 km and decreases to less than 0.5 m/s near the surface. Several of the slower falling dropsondes initially fall at the velocity of the faster dropsondes and suddenly slow to the rate of the slower sondes. This effect is not well explained and possibly related to an incomplete inflation of the slower parachute after launch.

The fall rate difference between the two parachute manufacturers has no effect on any of the measured parameters. However, it does affect the derivation of the vertical wind speed. Unlike in previous campaigns, where a theoretical fall rate was used to calculate vertical winds^15,17^, here the vertical wind speed is derived using the difference of the measured fall rate and the median fall rate over all soundings using each of the two parachute manufacturers. This method is virtually identical to using the model fall rate for the faster parachutes, but better captures the unexplained behavior of the slower parachutes. Vertical wind speeds above 6 km are less reliable and should not be used due to the unexplained behavior of the slower parachutes. Below that the statistical uncertainty in the vertical wind is consistent with published estimates^17^ and better than 0.6 m/s at a one sigma confidence level.

In six sondes (Table 9), the parachute did not deploy properly, and the sondes fell significantly faster than normal. Sounding 20210628_132740 produced a complete profile of all parameters and sounding 20210519_140632 produced a partial profile with data reaching down to 200 m above the ocean surface. For both soundings, the calculation of the vertical wind was disabled.

**Table 9:** Fast fall soundings.

#	RF	Sounding	Notes
1	RF36	20200921_163652	Telemetry lost soon after launch, ignored
2	RF46	20210220_164028	Telemetry lost soon after launch, ignored
3	RF58	20210330_134110	Telemetry lost soon after launch, ignored
4	RF67	20210519_140632	Partial profile
5	RF90	20210628_132740	Complete profile
6	RF101	20220111_200009	Telemetry lost soon after launch, ignored

The remaining soundings listed in Table 9 did not produce enough data because telemetry reception was lost soon after launch. These soundings have been left out of the dataset and are not considered further.

### Incomplete soundings

Two soundings produced only partial profiles. Sounding 20220302_203314 did not decode data in real time even though the computer clock was properly synchronized, leading to manual termination of data acquisition to troubleshoot the clock issue before the sonde reached the surface. All raw data were properly stored and allowed reprocessing down to 2454 m above the ocean surface. Sounding 20220118_140244 functioned normally down to 1672 m above the ocean surface, when telemetry transmission suddenly stopped.

Both profiles were retained in the data archive. Geopotential height calculation was initiated using the lowest GPS altitude, which may introduce an additional geopotential height error of up to 20 m.

### Aircraft temperature and relative humidity

The aircraft data system shared observations such as position, altitude, speed, ambient pressure, ambient temperature, and other parameters with the AVAPS data system. These data are shared in the Interagency Working Group standard format number 1 (IWG1), which was defined by the Interagency Working Group for Airborne Data and Telecommunications Systems (IWGADTS). The quality-controlled data store the atmospheric conditions as well as the location of the aircraft at the moment of sonde release as reference data.

The temperature included in the IWG1 data stream was not reported in °C and incorrectly stored in all AVAPS data files. The atmospheric temperature was reported by the aircraft data system as T + 512, where T is in °C. This offset was subtracted in quality-controlled data. The uncertainty of the ambient temperature reported by the aircraft data system is unknown and may be large but does not affect the dropsonde profile data.

Relative humidity was not reported by the aircraft data stream. The quality-controlled data contain a value of 20%, which allowed ASPEN to calculate a geopotential altitude at the top of the profile. This value has no effect on any of the dropsonde profile data.

## Data Records

The ACTIVATE dropsonde data are freely available at the NASA Langley Atmospheric Science Data Center^18^. The files are in the ICARTT format regularly used by NASA for field data. The data archive provides one file per dropsonde sounding. File names contain the associated campaign name (ACTIVATE), instrument (dropsonde), aircraft name, flight date and time information, and revision number. Each file contains the relevant metadata to identify the time and location of the observation along with other information relevant for data usage including the data principal investigator (PI) information for questions. The file name should not be used to identify the time of release and may be in conflict with the correct time stamp stored as part of the metadata (see above).

The soundings have also been archived at the NCAR/Earth Observing Laboratory^19^ using the NetCDF format and the Climate and Forecasting (http://cfconventions.org) metadata convention version 1.6.

## Technical Validation

### Sounding metrics

ACTIVATE focused on aerosol-cloud-meteorology interactions in marine boundary layer clouds over the northwest Atlantic region. Dropsondes were launched in both cloud-free and cloudy conditions. Marine boundary layer clouds ranged from stratiform to cumulus clouds, including both warm and mixed-phase clouds. Here, we show summary figures and statistics to demonstrate the reliability of this dropsonde type and to highlight the range of observations covered by the ACTIVATE dropsonde dataset.

Pressure, temperature, and humidity are transmitted twice per second, while horizontal winds are transmitted four times per second. Considering the time constants of the sensors, the fall rate, and the smoothing applied by ASPEN, the effective vertical resolution is in the range between 15 to 40 m, depending on altitude.

**Fig. 3:**
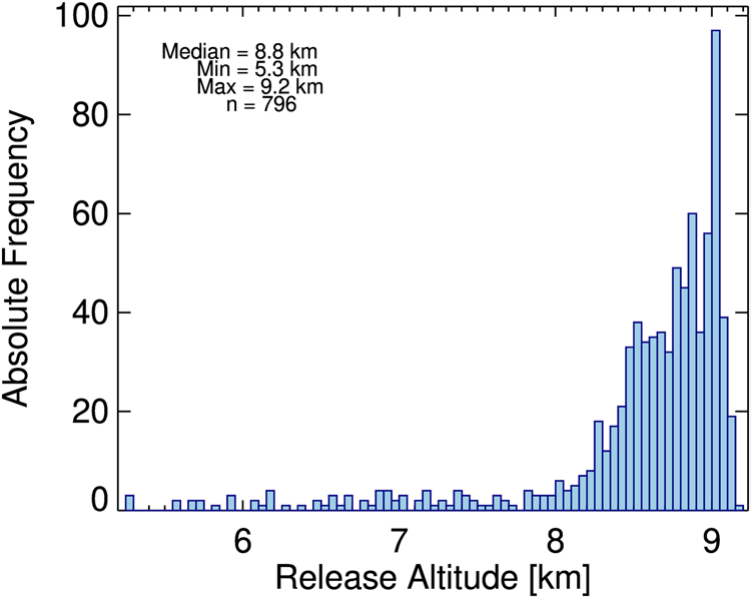
Distribution of the release altitudes during ACTIVATE. Sondes were predominantly launched at around 9 km. The lowest release altitude was 5.3 km and the highest 9.2 km.

Sondes were released at a median aircraft speed of 111 m/s and an altitude range between 5.3 and 9.2 km (Fig. 3). Dropsonde sensors require some time after release for equilibration to atmospheric conditions, which limits the effective ceiling altitude of the profiles roughly to 200 m below release altitude.

**Fig. 4:**
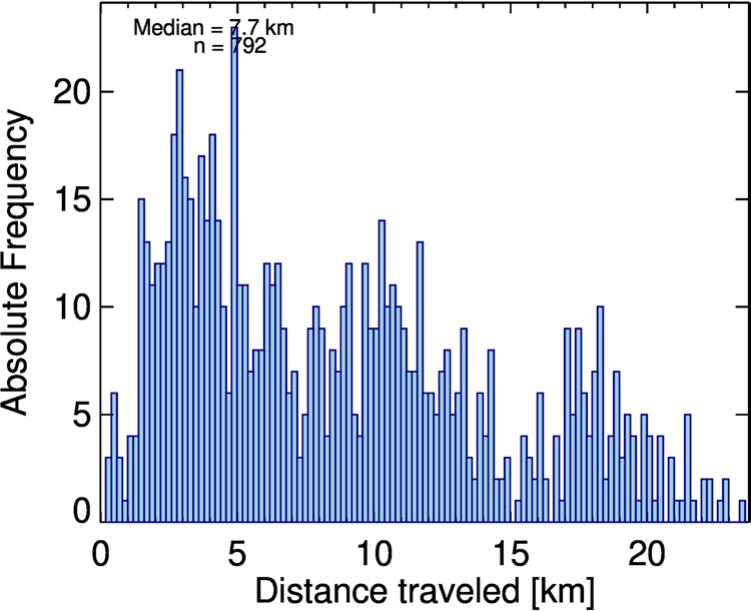
Histogram of the distance between launch and landing for all dropsondes during ACTIVATE.

Wind speeds during ACTIVATE were typically less than 20 m/s in the lower part of the profile and in some flights reached 80 m/s in the middle troposphere near the top of the profile. As a result, the horizontal drift of the dropsondes between release and landing is up to 23 km with a median drift of 7.7 km (Fig. 4).

**Fig. 5:**
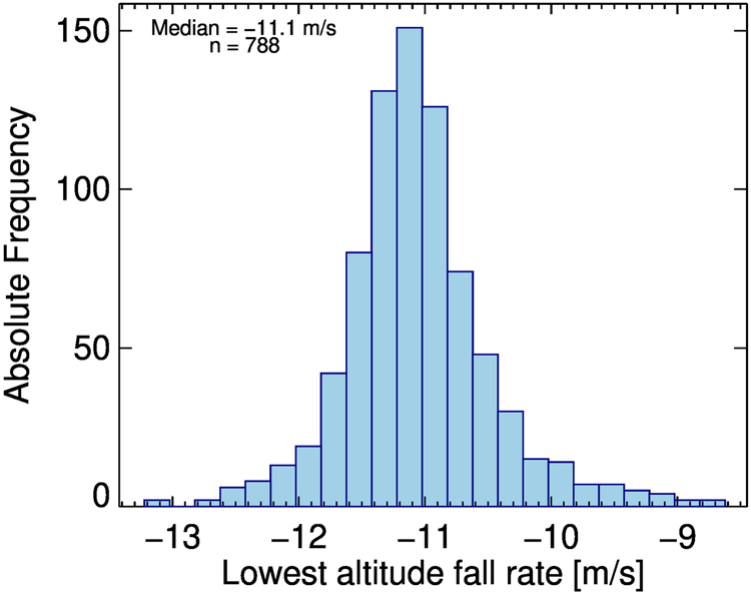
Distribution of the fall rates before landing. Soundings in which the parachute failed and which did not transmit data down to the surface are not included.

A histogram of the fall rate at landing is shown in Fig. 5. Soundings with parachute failure and early telemetry loss are excluded from this plot. The average fall time for all soundings is 11.1 ± 0.6 m/s. The statistics do not distinguish between the two different parachute manufacturers, which contributes to the slightly larger spread of fall rates at landing (see Fig. 2).

All housekeeping data are within their expected range. The battery voltage and the internal temperature for all sondes indicate that all sondes operated as expected and that no external factors related to the launch affected the measurements.

No sounding encountered updrafts, which may occur in strong deep convection, in particular in hurricane surveillance soundings.

### Atmospheric observations

**Fig. 6:**
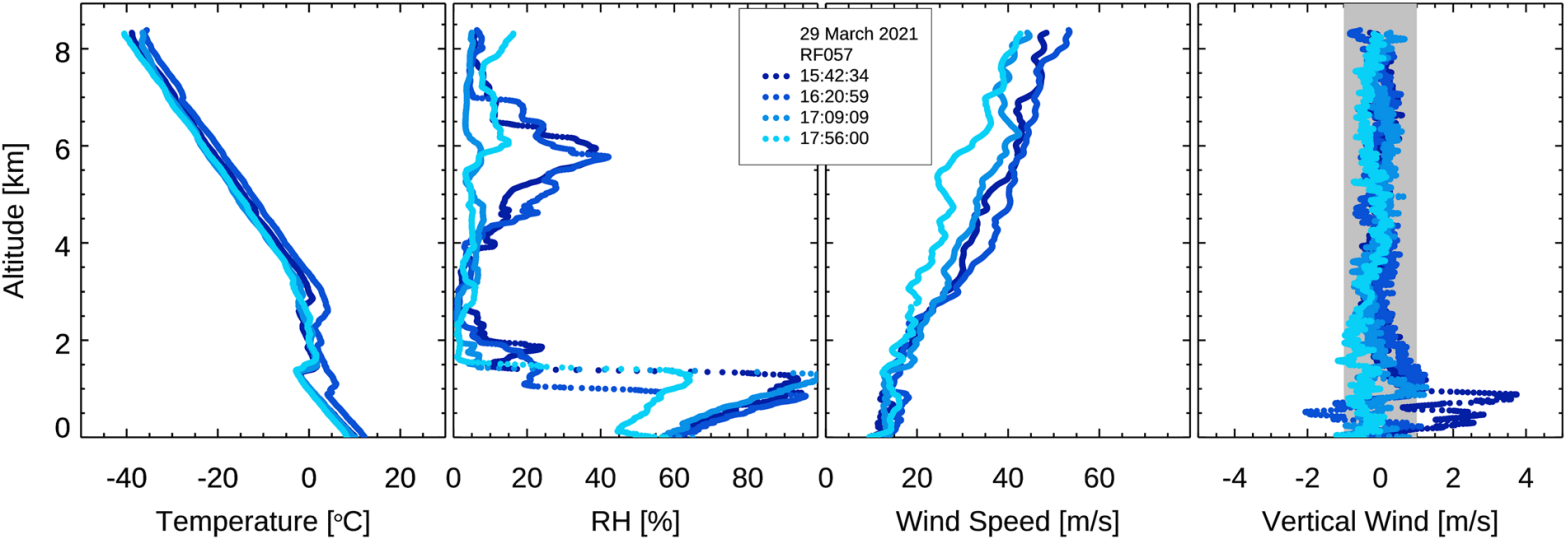
Vertical profiles from four soundings on 29 March 2021. The panels from left to right show air temperature, relative humidity (over liquid water), total wind speed, and vertical wind speed. The uncertainty of the vertical wind speed of 1 m/s is indicated by the grey shaded area.

Dropsondes report simultaneous profiles of pressure, temperature, relative humidity, and horizontal winds. A vertical wind component can be roughly estimated from variations of the fall rate of the dropsondes. During almost all research flights (i.e., “statistical survey flights”) typically between four and eight dropsondes were released, with high intensity research flights (i.e., “process study flights”) releasing up to 23 sondes in one flight. A simple example of the type of profiles that were generated is shown in Fig. 6. Research flight 57 released four soundings on 29 March 2021 within 135 min and over a distance of 450 km. The high accuracy of the observations allows for analysis of the atmospheric changes between the sonde releases.

**Fig. 7:**
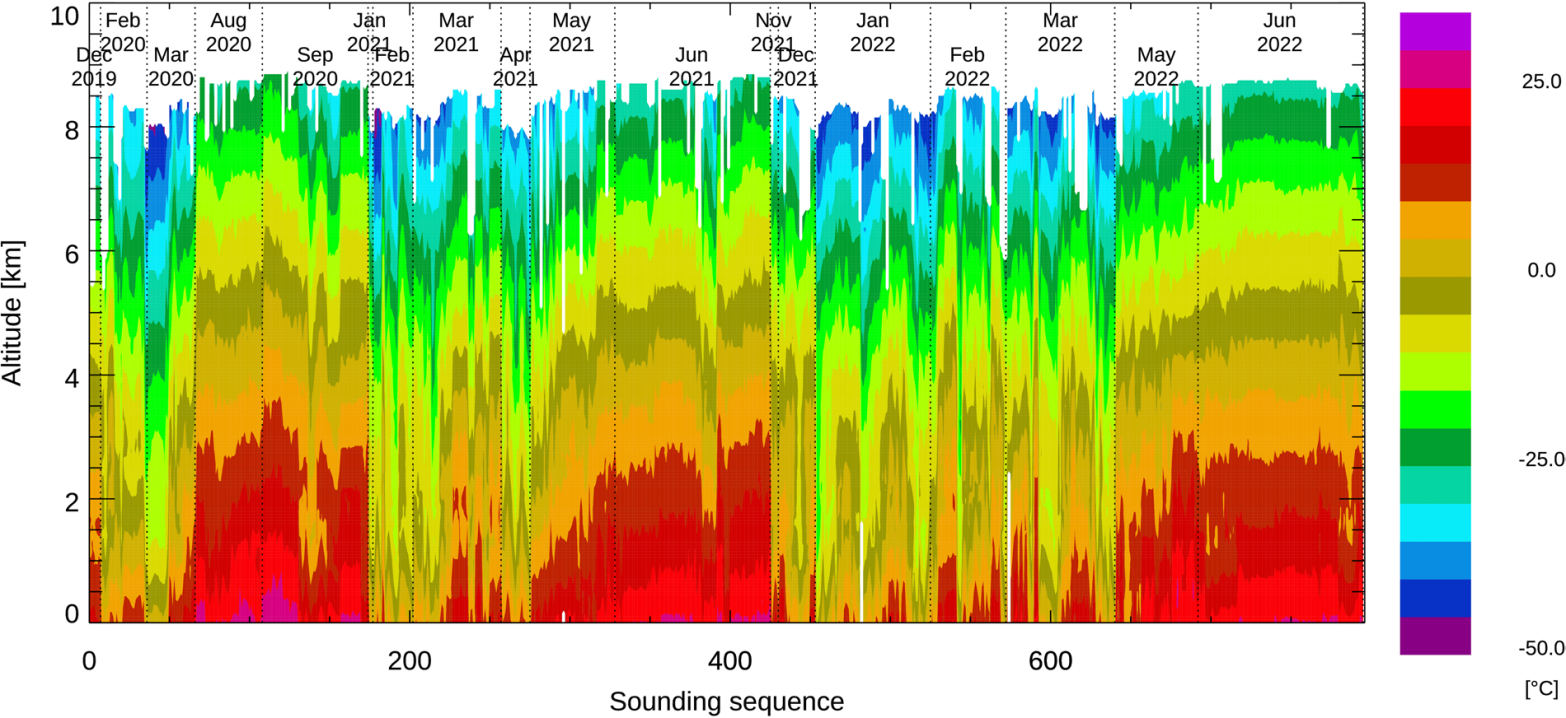
Color contours of air temperature from all soundings sequentially arranged. Missing data are shown in white. All soundings are shown in the sequence in which they were released and cover all deployments between February 2020 and June 2022. Deployment months are separated by vertical dotted lines; individual research flight numbers are not indicated.

The temperature measured by all dropsondes is shown as a contour plot in Fig. 7. The temperature at flight level was in the range of −13 °C to −47 °C depending on the aircraft altitude and time of year and in the range of −10 °C to 29 °C near the surface.

The surface pressure reported by the sondes is an extrapolation of the last measured air pressure above the surface to sea level using the current fall rate. The surface pressure reported by all sondes, which was transmitted to the surface, varied between 1000 hPa and 1040 hPa.

**Fig. 8:**
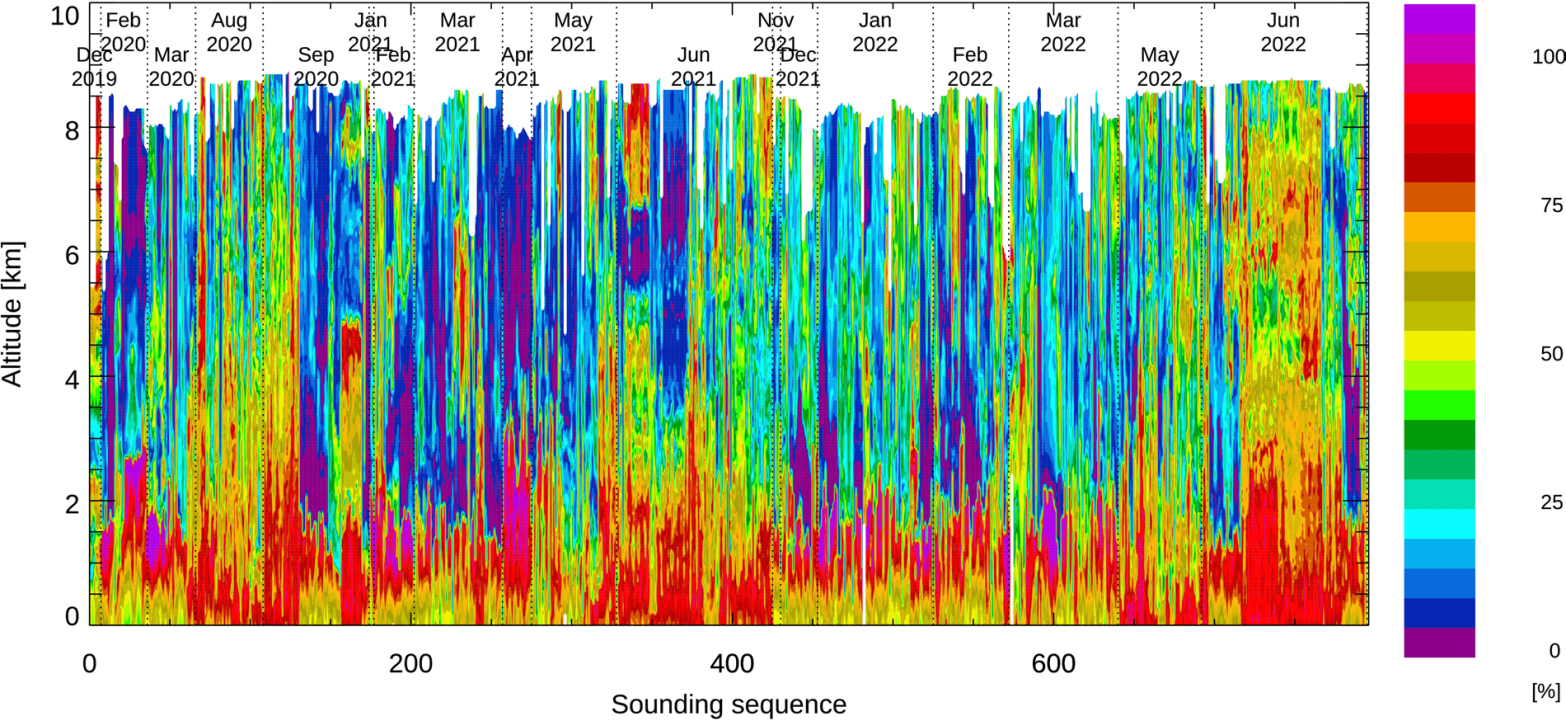
Color contours of relative humidity from all soundings sequentially arranged. Note that relative humidity is shown with respect to ice at temperatures below freezing (typically middle to upper troposphere).

**Fig. 9:**
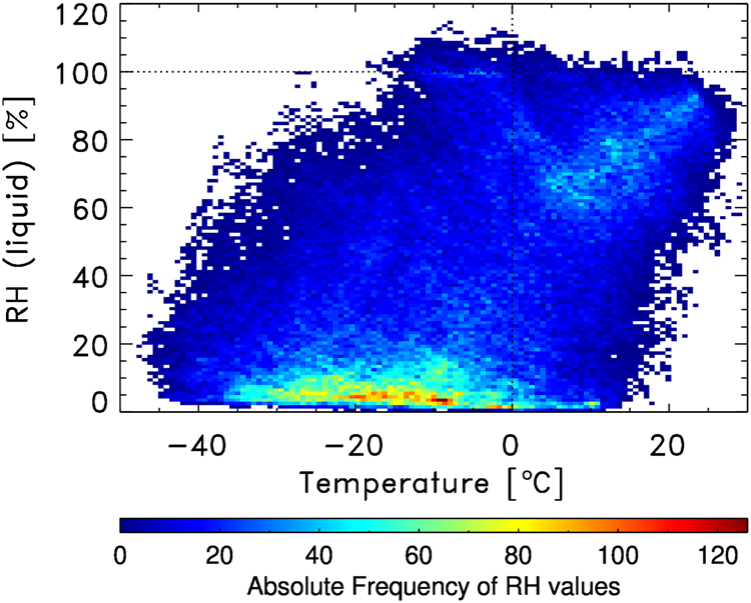
Distribution of relative humidity (liquid) over temperature for all soundings during ACTIVATE. The horizontal dashed line indicates saturation over liquid, while the vertical dashed line indicates the freezing temperature. Data bins are 1% relative humidity and 0.7 °C.

Relative humidity measured by all dropsondes is shown in Fig. 8. At temperatures below 0 °C, relative humidity is expressed as relative humidity over ice instead of the conventional relative humidity over liquid water. All relative humidity observations as a function of atmospheric temperature are shown as a density plot in Fig. 9. Here we used the unfiltered and unsmoothed raw data to provide an indication for the behavior and accuracy of the NRD41 humidity sensor at high relative humidity. Between −15 °C and −5 °C, the most common reported relative humidity is 101%, consistent with saturation in supercooled liquid clouds. The small bias of 1% is well within the uncertainty of the humidity sensor^20^ and consistent with ground checks of relative humidity under saturated conditions^21^ done within the Global Climate Observing System Reference Upper Air Network (GRUAN). A small number of soundings reported relative humidity maxima up to 115%. Instrumental noise, prior to filtering and smoothing and the likely presence of supercooled liquid cloud particles contribute to these rare extreme values. In the quality controlled dataset, all relative humidity values above 100% are set to 100%. These values are a strong indication for the presence of clouds.

**Fig. 10:**
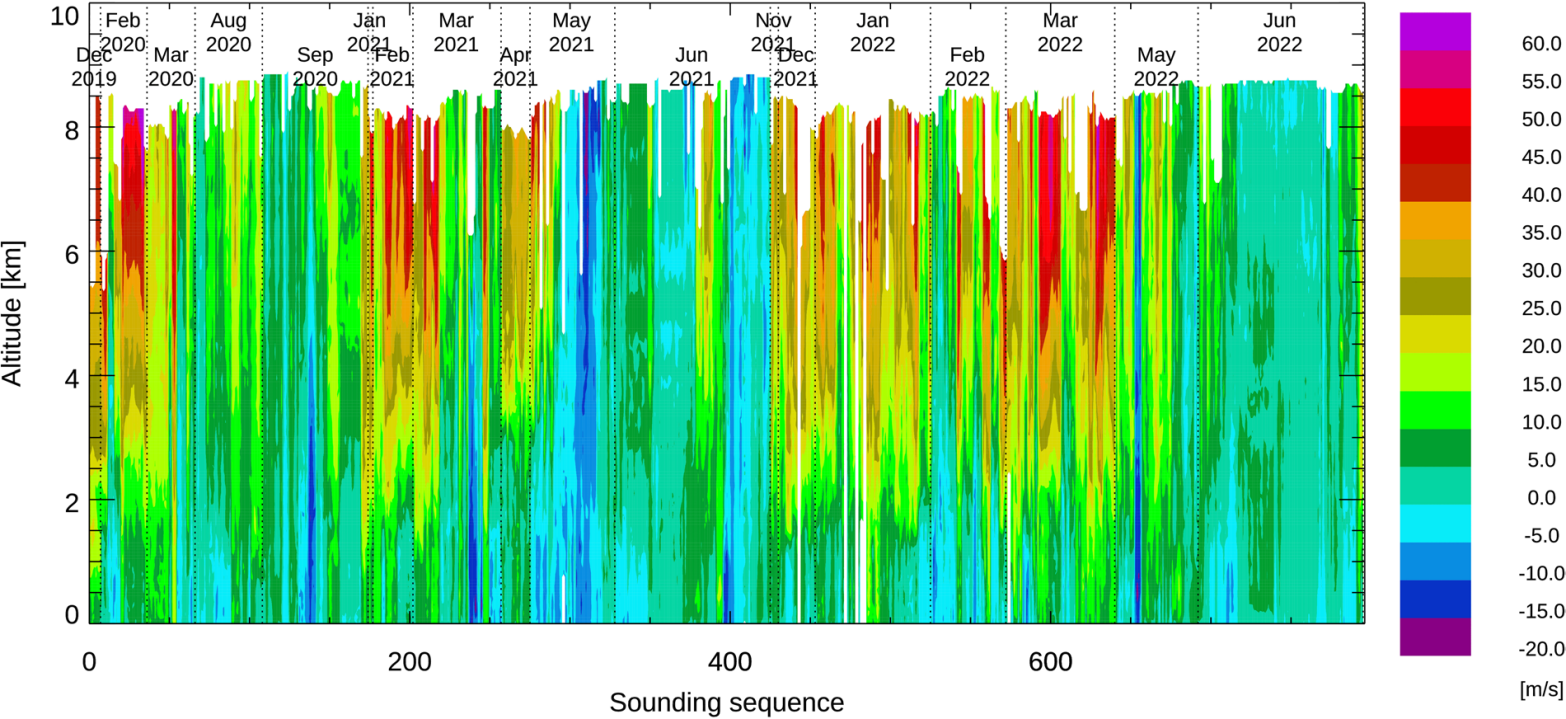
Color contours showing zonal wind speed measurements from all soundings sequentially arranged.

Zonal wind speeds are shown in Fig. 10. Brown colors indicate westerly winds, while green and blue colors indicate easterly winds. Data files contain the north and eastward wind components as well as wind speed and wind direction. Below 2 km, no sounding reported wind speeds above 25 m/s. The jet stream dominates the wind profiles in most soundings between the middle of September and middle of May. The soundings during the summer months are not affected by the jet stream except the soundings between 15 and 18 June 2021.

**Fig. 11:**
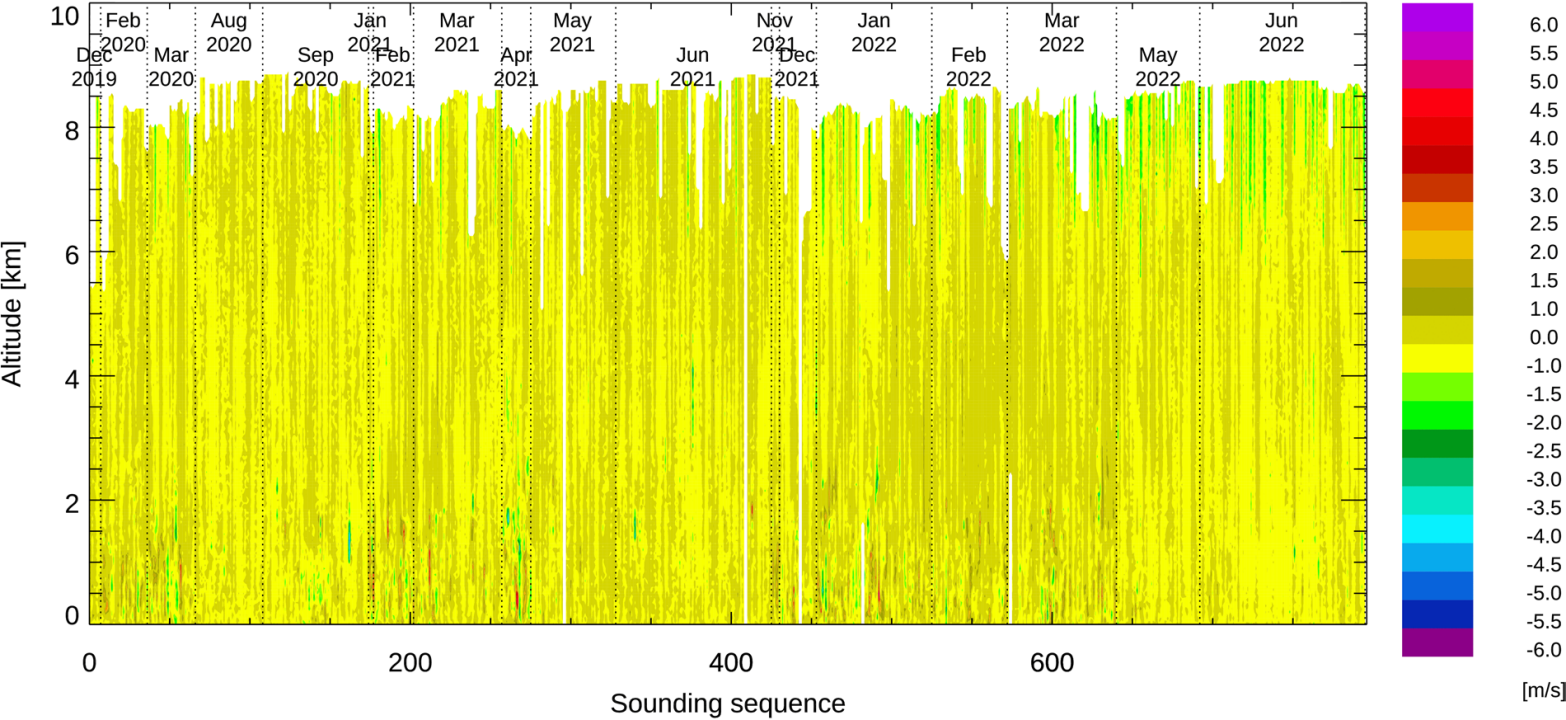
Vertical wind speed derived from the fall rate of the dropsondes.

The vertical wind speeds derived from the fall rate of the dropsondes are shown in Fig. 11. Vertical wind speed estimates for the two fast-fall sondes and for short periods during which the parachute had not yet fully inflated were set to missing.

The overall estimate of the vertical wind speed uncertainty is less than 1 m/s, except for altitudes above 6 km, where some parachutes showed unexplained production variability. Vertical wind speeds above 6 km are left in the dataset but should generally be treated with great caution. Areas of vertical updraft and downdraft larger than 1 m/s can be identified using this vertical wind speed estimate. Below 4 km, numerous soundings show vertical wind speeds up to 2 m/s and some show layers of vertical wind speeds of up to 6 m/s.

## Data Availability

The ASPEN software package and a description of its functionality are available at https://www.eol.ucar.edu/content/aspen.
