# Publisher Correction: Dropsonde observations during the Aerosol Cloud meTeorology Interactions oVer the western ATlantic Experiment

**DOI:** 10.1038/s41597-023-02774-z

**Published:** 2023-12-18

**Authors:** Holger Vömel, Armin Sorooshian, Claire Robinson, Taylor J. Shingler, Kenneth Lee Thornhill, Luke D. Ziemba

**Affiliations:** 1 National Center for Atmospheric Research, Boulder, CO 30301 USA; 2 Department of Chemical and Environmental Engineering, University of Arizona, Tucson, AZ 85721 USA; 3 NASA Langley Research Center, Hampton, VA 23681 USA

**Keywords:** Atmospheric chemistry, Atmospheric dynamics, Atmospheric chemistry, Atmospheric dynamics

Correction to: *Scientific Data* 10.1038/s41597-023-02647-5, published online 01 November 2023

Cell highlighting that was originally included in Table 1 was not typeset in the original version. This has been replaced with indicators (*) against the relevant data points so that these labels are now shown in the HTML and pdf versions of the article. The table caption has also been updated to reflect this change, with the phrase “Grey shaded research flights contain soundings with at least one issue discussed in the following paragraphs” replaced with “A * following the number of dropsonde launches indicates research flights containing soundings with at least one issue discussed in the following paragraphs.”

